# Supplementary material for: NanoDefiner Framework and e-Tool Revisited According to the European Commission’s Nanomaterial Definition 2022/C 229/01
Source: Nanomaterials (Basel). 2023 Mar 9;13(6):990. doi: 10.3390/nano13060990 (PMC10056892; doi:10.3390/nano13060990)
Supplement: Supplementary file 1 [file nanomaterials-13-00990-s001.zip › s2_IRMM-387_etool_report.pdf]

Dossier Report: Usecase IRMM387 BaSO4 ultrafine

Generated by the NanoDefiner e-tool which is part of the NanoDefine project. See http://nanodefine.eu for more information. Any recommendations given and results determined by the NanoDefiner are supplied without liability.

Dossier information

Name	Usecase IRMM387 BaSO4 ultrafine
Comment	–

Sample information

Sample tested	Usecase IRMM387 BaSO4 ultrafine mono sample
Date	2023-02-06 08:59:16
Institution	FH Dortmund
Person	Dr. Philipp Müller
Multi-type substance	No

Particle type information

Particle type #1 based on [IRMM-387] BaSO4, ultrafine grade

Comment: –
Particle type description completeness: 81%

Release of ions, atoms, molecues	No
Shape	Sphere or similar
Chemical composition	Inorganic
Particle size range	[10,150]
Magnetism	Unknown
Agglomeration	Yes
Multimodality	Unknown
Aggregation	Yes
Polydispersity	Highly polydisperse
Composites	None
Electron beam sensitivity	No
Conductivity	Insulator
Vacuum sensitivity	No
Fluorescence	Unknown
Stable temperature	[-273,500]
Functionalisation	No
Dispersibility	Dispersible in polar liquids<br>Dispersible in non-polar liquids<br>Dispersible in material-specific media<br>Dispersible in aqueous media<br>Can be aerosolized
Trade form	Powder
Presence of different sized particles	No
Group	
Light absorption	Unknown
Dimension	3 small dimensions

Method information

DLS

Tier	Tier 1 (screening)
Comment	–
Preparation	Creation of a dispersion
Preparation comment	–
Measurement technique description completeness	99%
Weighted measurement technique description completeness	100%
Data format	Manual D_{50}
Result	76nm
Measurement technique uncertainty	Unknown
Argumentation	D_{50}

Decision: Categorised as **nanomaterial**

Warning: Method results may be unreliable due to the following particle type attributes being unknown.

Attribute	Measurement technique value
Multimodality	Multimodality support:<br>Moderate distance between peaks<br>Peak size differ > factor 10

BET

Tier	Tier 1 (screening)
Comment	–
Preparation	Sample drying
Preparation comment	–
Measurement technique description completeness	96%
Weighted measurement technique description completeness	100%
Data format	Manual D_{50}
Result	149nm
Measurement technique uncertainty	Unknown
Argumentation	D_{50}

Decision: Categorised as **borderline material**

Warning: This measurement technique may not yield reliable results if the material to be analysed shows porous properties or severe states of aggregation/agglomeration. As per the updated EC nanomaterial definition recommendation, the particle type shape is no longer a crucial criterion for the analysis. A VSSA 6 will result in a non-nanomaterial decision while a VSSA above or equal to this threshold suggests a potential nanomaterial for which further analysis is suggested.

sprayDEMA

Tier	Tier 1 (screening)
Comment	–
Preparation	None
Preparation comment	–
Measurement technique description completeness	99%
Weighted measurement technique description completeness	100%
Data format	Manual D_{50}
Result	53nm
Measurement technique uncertainty	Unknown
Argumentation	D_{50}

Decision: Categorised as **nanomaterial**

AC-turb

Tier	Tier 1 (screening)
Comment	disc
Preparation	Creation of a dispersion
Preparation comment	–
Measurement technique description completeness	99%
Weighted measurement technique description completeness	100%
Data format	Manual D_{50}
Result	66nm
Measurement technique uncertainty	Unknown
Argumentation	D_{50}

Decision: Categorised as **nanomaterial**

AC-turb

Tier	Tier 1 (screening)
Comment	cuvette
Preparation	Creation of a dispersion
Preparation comment	–
Measurement technique description completeness	99%
Weighted measurement technique description completeness	100%
Data format	Manual D_{50}
Result	43nm
Measurement technique uncertainty	Unknown
Argumentation	D_{50}

Decision: Categorised as **nanomaterial**

AC-RI

Tier	Tier 1 (screening)
Comment	cuvette
Preparation	Creation of a dispersion
Preparation comment	–
Measurement technique description completeness	99%
Weighted measurement technique description completeness	100%
Data format	Manual D_{50}
Result	24nm
Measurement technique uncertainty	Unknown
Argumentation	D_{50}

Decision: Categorised as **nanomaterial**

SEM

Tier	Tier 2 (confirmatory)
Comment	–
Preparation	Creation of a substrate
Preparation comment	–
Measurement technique description completeness	99%
Weighted measurement technique description completeness	100%
Data format	Manual D_{50}
Result	35nm
Measurement technique uncertainty	Unknown
Argumentation	D_{50}

Decision: Categorised as **nanomaterial**

miniTEM-T2

Tier	Tier 2 (confirmatory)
Comment	–
Preparation	Creation of a substrate
Preparation comment	–
Measurement technique description completeness	99%
Weighted measurement technique description completeness	100%
Data format	Manual D_{50}
Result	31nm
Measurement technique uncertainty	Unknown
Argumentation	D_{50}

Decision: Categorised as **nanomaterial**

Warning: Performance comparable to standard TEM (Tier 2) if sample preparation and image evaluation is optimised for each material.

Acknowledgements

The NanoDefine project has received funding from the European Union’s Seventh Programme for research, technological development and demonstration under grant agreement No 604347.

Appendix

Glossary

Particle type description completeness

The measure C^m describes the percentage presence of completeness in the particle type description and will be present in the dossier report. The more known particle type properties are given, the more certain measurement technique recommendations will be. Hence, it is highly desireable to keep C^m high.

$C^m < 50\%$ = Low completeness in the particle type description
$C^m \geq 50\%$ = Moderate completeness in the particle type description
$C^m > 80\%$ = High completeness in the particle type description

Weighted particle type description completeness

The measure $\hat{C}^m$ describes a weighted pendant of C^m and will be present in the dossier report. The measurement technique-individual weight of a particle type property adjusts its individual impact on the measure. For instance, given that a measurement technique profile weights a certain particle type property 50 % instead of 100 %, it only has half the impact on $\hat{C}^m$ than it has on the unweighted measure C^m. Given that a particle type property is weighted 0 %, stating it will have no impact on $\hat{C}^m$. This allows to lower or eliminate the impact of unknown values for specific material properties that are less or not relevant for a measurement technique. Given a low C^m also a moderate to high $\hat{C}^m$ is possible. A high $\hat{C}^m$ is generally more desireable than a high C^m due to fulfilled description of more relevant properties. Please note: $\hat{C}^m$ equals C^m for default measurement technique profiles in the knowledge base.

Value interpretation for Ct and Ĉt can be adopted from intervals given for Cm and Ĉm.

$\hat{C}^m < 50\%$ = Low weighted completeness in the particle type description for a specific measurement techique
$\hat{C}^m \geq 50\%$ = Moderate weighted completeness in the particle type description for a specific measurement techique
$\hat{C}^m > 80\%$ = High weighted completeness in the particle type description for a specific measurement techique

Measurement technique description completeness

The measure C^t described the percentage presence of completeness in the measurement technique description in the knowledge base and will be present in the dossier report. The more described properties are given for a measurement technique, the more certain its recommendations will be. Hence, it is highly desireable to keep C^t high.

The measure $\hat{C}^t$ described the weighted pendant of C^t and will be present in the dossier report. The measurement technique-individual weight of a measurement technique property adjusts its individual impact on the measure. $\hat{C}^t$ works analog to $\hat{C}^m$, but is focused on the relevance of measurement technique properties. For further information see the description of Ĉm. Please note: $\hat{C}^t$ equals C^t for default measurement technique profiles in the knowledge base.

Measurement technique overview

Measurement technique	Tier	Available	Suitable	Ĉ^t	C^t
AC-RI	Tier 1 (screening)	Yes	Yes	99%	100%
AC-turb	Tier 1 (screening)	Yes	Yes	99%	100%
BET	Tier 1 (screening)	Yes	Yes	96%	100%
DLS	Tier 1 (screening)	Yes	Yes	99%	100%
miniTEM-T1	Tier 1 (screening)	Yes	Yes	99%	100%
sprayDEMA	Tier 1 (screening)	Yes	Yes	99%	100%
miniTEM-T2	Tier 2 (confirmatory)	Yes	Yes	99%	100%
SEM	Tier 2 (confirmatory)	Yes	Yes	99%	100%
TEM	Tier 2 (confirmatory)	Yes	Yes	99%	100%
AFM	Potential tier 2 (not assessed)	Yes	Yes	99%	100%

Unsuitability details
