# Supplementary material for: NanoDefiner Framework and e-Tool Revisited According to the European Commission’s Nanomaterial Definition 2022/C 229/01
Source: Nanomaterials (Basel). 2023 Mar 9;13(6):990. doi: 10.3390/nano13060990 (PMC10056892; doi:10.3390/nano13060990)
Supplement: Supplementary file 1 [file nanomaterials-13-00990-s001.zip › s3_IRMM-380_etool_report.pdf]

NanoDefiner Test Service

Institution logo can be configured here

Dossier Report: Usecase IRMM-380 pigment yellow 83 nano

Generated by the NanoDefiner e-tool which is part of the NanoDefine project. See http://nanodefine.eu for more information. Any recommendations given and results determined by the NanoDefiner are supplied without liability.

Dossier information

Name	Usecase IRMM-380 pigment yellow 83 nano
Comment	–

Sample information

Sample tested	Usecase IRMM-380 pigment yellow 83 nano mono sample
Date	2023-02-06 09:03:21
Institution	FH Dortmund
Person	Dr. Philipp Müller
Multi-type substance	No

Particle type information

Particle type #1 based on [IRMM-380] Organic pigment yellow 83, nano grade

Comment: –
Particle type description completeness: 81%

Release of ions, atoms, molecues	No
Shape	Tubes, fibres, rods (length:ø ≥ 3)
Chemical composition	Organic, particulate
Particle size range	[20,90]
Magnetism	Unknown
Aggregation	Yes
Agglomeration	Yes
Multimodality	Unknown
Polydispersity	Highly polydisperse
Composites	None
Electron beam sensitivity	Yes
Vacuum sensitivity	No
Conductivity	Insulator
Fluorescence	Unknown
Stable temperature	[-273,100]
Dispersibility	Dispersible in polar liquids<br>Dispersible in non-polar liquids<br>Dispersible in material-specific media<br>Dispersible in aqueous media<br>Can be aerosolized
Functionalisation	No
Trade form	Powder
Presence of different sized particles	No
Group	
Light absorption	Unknown
Dimension	2 small dimensions

Method information

BET

Tier	Tier 1 (screening)
Comment	–
Preparation	Sample drying
Preparation comment	–
Measurement technique description completeness	96%
Weighted measurement technique description completeness	100%
Data format	VSSA value
Result	–
Measurement technique uncertainty	Unknown
VSSA value	93.46
Argumentation	For the given particle type and VSSA value, no nano/non-nano decision can be made, please use another method (for regulatory purposes, using a tier 2 method is recommended)

Decision: No decision could be reached, see argumentation above

Warning: This measurement technique may not yield reliable results if the material to be analysed shows porous properties or severe states of aggregation/agglomeration. As per the updated EC nanomaterial definition recommendation, the particle type shape is no longer a crucial criterion for the analysis. A VSSA 6 will result in a non-nanomaterial decision while a VSSA above or equal to this threshold suggests a potential nanomaterial for which further analysis is suggested.

AC-turb-disc

Tier	Tier 1 (screening)
Comment	–
Preparation	Creation of a dispersion
Preparation comment	–
Measurement technique description completeness	99%
Weighted measurement technique description completeness	100%
Data format	Manual D_{50}
Result	52nm
Measurement technique uncertainty	Unknown
Argumentation	D_{50}

Decision: Categorised as **nanomaterial**

Warning: Measurement technique was chosen despite being unsuitable for the material. See the appendix for additional details.

AC-turb-cuvette

Tier	Tier 1 (screening)
Comment	–
Preparation	Creation of a dispersion
Preparation comment	–
Measurement technique description completeness	99%
Weighted measurement technique description completeness	100%
Data format	Manual D_{50}
Result	34nm
Measurement technique uncertainty	Unknown
Argumentation	D_{50}

Decision: Categorised as **nanomaterial**

Warning: Measurement technique was chosen despite being unsuitable for the material. See the appendix for additional details.

miniTEM-T2

Tier	Tier 2 (confirmatory)
Comment	–
Preparation	Creation of a dispersion
Preparation comment	–
Measurement technique description completeness	99%
Weighted measurement technique description completeness	100%
Data format	Manual D_{50}
Result	48nm
Measurement technique uncertainty	Unknown
Argumentation	D_{50}

Decision: Categorised as **nanomaterial**

Warning: Measurement technique was chosen despite being unsuitable for the material. See the appendix for additional details.

Warning: Performance comparable to standard TEM (Tier 2) if sample preparation and image evaluation is optimised for each material.

SEM

Tier	Tier 2 (confirmatory)
Comment	–
Preparation	None
Preparation comment	–
Measurement technique description completeness	99%
Weighted measurement technique description completeness	100%
Data format	Manual D_{50}
Result	40nm
Measurement technique uncertainty	Unknown
Argumentation	D_{50}

Decision: Categorised as **nanomaterial**

Warning: Measurement technique was chosen despite being unsuitable for the material. See the appendix for additional details.

DLS

Tier	Tier 1 (screening)
Comment	–
Preparation	Creation of a dispersion
Preparation comment	–
Measurement technique description completeness	99%
Weighted measurement technique description completeness	100%
Data format	Manual D_{50}
Result	68nm
Measurement technique uncertainty	Unknown
Argumentation	D_{50}

Decision: Categorised as **nanomaterial**

Warning: Measurement technique was chosen despite being unsuitable for the material. See the appendix for additional details.

Warning: Method results may be unreliable due to the following particle type attributes being unknown.

Attribute	Measurement technique value
Multimodality	Multimodality support:<br>Moderate distance between peaks<br>Peak size differ > factor 10

Acknowledgements

The NanoDefine project has received funding from the European Union’s Seventh Programme for research, technological development and demonstration under grant agreement No 604347.

Appendix

Glossary

Particle type description completeness

The measure C^m describes the percentage presence of completeness in the particle type description and will be present in the dossier report. The more known particle type properties are given, the more certain measurement technique recommendations will be. Hence, it is highly desireable to keep C^m high.

$C^m < 50\%$ = Low completeness in the particle type description
$C^m \geq 50\%$ = Moderate completeness in the particle type description
$C^m > 80\%$ = High completeness in the particle type description

Weighted particle type description completeness

The measure $\hat{C}^m$ describes a weighted pendant of C^m and will be present in the dossier report. The measurement technique-individual weight of a particle type property adjusts its individual impact on the measure. For instance, given that a measurement technique profile weights a certain particle type property 50 % instead of 100 %, it only has half the impact on $\hat{C}^m$ than it has on the unweighted measure C^m. Given that a particle type property is weighted 0 %, stating it will have no impact on $\hat{C}^m$. This allows to lower or eliminate the impact of unknown values for specific material properties that are less or not relevant for a measurement technique. Given a low C^m also a moderate to high $\hat{C}^m$ is possible. A high $\hat{C}^m$ is generally more desireable than a high C^m due to fulfilled description of more relevant properties. Please note: $\hat{C}^m$ equals C^m for default measurement technique profiles in the knowledge base.

Value interpretation for Ct and Ĉt can be adopted from intervals given for Cm and Ĉm.

$\hat{C}^m < 50\%$ = Low weighted completeness in the particle type description for a specific measurement techique
$\hat{C}^m \geq 50\%$ = Moderate weighted completeness in the particle type description for a specific measurement techique
$\hat{C}^m > 80\%$ = High weighted completeness in the particle type description for a specific measurement techique

Measurement technique description completeness

The measure C^t described the percentage presence of completeness in the measurement technique description in the knowledge base and will be present in the dossier report. The more described properties are given for a measurement technique, the more certain its recommendations will be. Hence, it is highly desireable to keep C^t high.

The measure $\hat{C}^t$ described the weighted pendant of C^t and will be present in the dossier report. The measurement technique-individual weight of a measurement technique property adjusts its individual impact on the measure. $\hat{C}^t$ works analog to $\hat{C}^m$, but is focused on the relevance of measurement technique properties. For further information see the description of Ĉm. Please note: $\hat{C}^t$ equals C^t for default measurement technique profiles in the knowledge base.

Measurement technique overview

Measurement technique	Tier	Available	Suitable	Ĉ^t	C^t
AC-RI	Tier 1 (screening)	Yes	No	99%	100%
AC-turb	Tier 1 (screening)	Yes	No	99%	100%
BET	Tier 1 (screening)	Yes	Yes	96%	100%
DLS	Tier 1 (screening)	Yes	No	99%	100%
miniTEM-T1	Tier 1 (screening)	Yes	No	99%	100%
sprayDEMA	Tier 1 (screening)	Yes	No	99%	100%
miniTEM-T2	Tier 2 (confirmatory)	Yes	No	99%	100%
SEM	Tier 2 (confirmatory)	Yes	No	99%	100%
TEM	Tier 2 (confirmatory)	Yes	No	99%	100%
AFM	Potential tier 2 (not assessed)	Yes	Yes	99%	100%

Unsuitability details

AC-RI

Name	Analytical Centrifugation, refractive index
Tier	Tier 1 (screening)
Measurement technique description completeness	99%
Weighted measurement technique description completeness	100%

Particle type #1 based on [IRMM-380] Organic pigment yellow 83, nano grade

Weighted particle type description completeness	82%

Attribute	Measurement technique	Particle type
Shape	Shape support:<br>Equiaxial<br>Sphere or similar	Shape:<br>Tubes, fibres, rods (length:ø ≥ 3)
Explanation:<br>The expected particle shape of the particulate component is not supported by the measurement technique.		
Dimension	Dimension support:<br>3 small dimensions	Dimension:<br>2 small dimensions
Explanation:<br>The expected number of small dimensions of the particulate component is not supported by the measurement technique.		
Trade form	Trade form support:<br>Suspension	Trade form:<br>Powder
Explanation:<br>The trade form of the particulate component is not supported by the measurement technique. However, given that at least one of the expected dispersibility options of the particulate component is supported by the measurement technique, this circumstance does not affect its recommendation.		

AC-turb

Name	Analytical Centrifugation, turbidity
Tier	Tier 1 (screening)
Measurement technique description completeness	99%
Weighted measurement technique description completeness	100%

Particle type #1 based on [IRMM-380] Organic pigment yellow 83, nano grade

Weighted particle type description completeness	82%

Attribute	Measurement technique	Particle type
Shape	Shape support:<br>Equiaxial<br>Sphere or similar	Shape:<br>Tubes, fibres, rods (length:ø ≥ 3)
Explanation:<br>The expected particle shape of the particulate component is not supported by the measurement technique.		
Dimension	Dimension support:<br>3 small dimensions	Dimension:<br>2 small dimensions
Explanation:<br>The expected number of small dimensions of the particulate component is not supported by the measurement technique.		
Trade form	Trade form support:<br>Suspension	Trade form:<br>Powder
Explanation:<br>The trade form of the particulate component is not supported by the measurement technique. However, given that at least one of the expected dispersibility options of the particulate component is supported by the measurement technique, this circumstance does not affect its recommendation.		

DLS

Name	Dynamic Light Scattering
Tier	Tier 1 (screening)
Measurement technique description completeness	99%
Weighted measurement technique description completeness	100%

Particle type #1 based on [IRMM-380] Organic pigment yellow 83, nano grade

Weighted particle type description completeness	82%

Attribute	Measurement technique	Particle type
Shape	Shape support:<br>Equiaxial<br>Sphere or similar	Shape:<br>Tubes, fibres, rods (length:ø ≥ 3)
Explanation:<br>The expected particle shape of the particulate component is not supported by the measurement technique.		
Dimension	Dimension support:<br>3 small dimensions	Dimension:<br>2 small dimensions
Explanation:<br>The expected number of small dimensions of the particulate component is not supported by the measurement technique.		
Trade form	Trade form support:<br>Suspension	Trade form:<br>Powder
Explanation:<br>The trade form of the particulate component is not supported by the measurement technique. However, given that at least one of the expected dispersibility options of the particulate component is supported by the measurement technique, this circumstance does not affect its recommendation.		

miniTEM-T1

Name	mini Transmission Electron Microscopy, Tier 1 settings
Tier	Tier 1 (screening)
Measurement technique description completeness	99%
Weighted measurement technique description completeness	100%

Particle type #1 based on [IRMM-380] Organic pigment yellow 83, nano grade

Weighted particle type description completeness	94%

Attribute	Measurement technique	Particle type
Electron beam sensitivity	Use of electron beam: Yes	Electron beam sensitivity: Yes

Explanation:

Degradation of the particulate component is may occur, as either the particulate component is expected to be sensitive to electron radiation and the measurement technique does apply electron radiation.

sprayDEMA

Name	Spray Differential Electrical Mobility Analysis
Tier	Tier 1 (screening)
Measurement technique description completeness	99%
Weighted measurement technique description completeness	100%

Particle type #1 based on [IRMM-380] Organic pigment yellow 83, nano grade

Weighted particle type description completeness	82%

Attribute	Measurement technique	Particle type
Shape	Shape support:<br>Equiaxial<br>Sphere or similar	Shape:<br>Tubes, fibres, rods (length:ø ≥ 3)
Explanation:<br>The expected particle shape of the particulate component is not supported by the measurement technique.		
Dimension	Dimension support:<br>3 small dimensions	Dimension:<br>2 small dimensions
Explanation:<br>The expected number of small dimensions of the particulate component is not supported by the measurement technique.		
Trade form	Trade form support:<br>Suspension	Trade form:<br>Powder
Explanation:<br>The trade form of the particulate component is not supported by the measurement technique. However, given that at least one of the expected dispersibility options of the particulate component is supported by the measurement technique, this circumstance does not affect its recommendation.		

miniTEM-T2

Name	mini Transmission Electron Microscopy, Tier 2 settings
Tier	Tier 2 (confirmatory)
Measurement technique description completeness	99%
Weighted measurement technique description completeness	100%

Particle type #1 based on [IRMM-380] Organic pigment yellow 83, nano grade

Weighted particle type description completeness	94%

Attribute	Measurement technique	Particle type
Electron beam sensitivity	Use of electron beam: Yes	Electron beam sensitivity: Yes

Explanation:

Degradation of the particulate component is may occur, as either the particulate component is expected to be sensitive to electron radiation and the measurement technique does apply electron radiation.

SEM

Name	Scanning Electron Microscopy
Tier	Tier 2 (confirmatory)
Measurement technique description completeness	99%
Weighted measurement technique description completeness	100%

Particle type #1 based on [IRMM-380] Organic pigment yellow 83, nano grade

Weighted particle type description completeness	82%

Attribute	Measurement technique	Particle type
Electron beam sensitivity	Use of electron beam: Yes	Electron beam sensitivity: Yes

Explanation:

Degradation of the particulate component is may occur, as either the particulate component is expected to be sensitive to electron radiation and the measurement technique does apply electron radiation.

TEM

Name	Transmission Electron Microscopy
Tier	Tier 2 (confirmatory)
Measurement technique description completeness	99%
Weighted measurement technique description completeness	100%

Particle type #1 based on [IRMM-380] Organic pigment yellow 83, nano grade

Weighted particle type description completeness	94%

Attribute	Measurement technique	Particle type
Electron beam sensitivity	Use of electron beam: Yes	Electron beam sensitivity: Yes

Explanation:

Degradation of the particulate component is may occur, as either the particulate component is expected to be sensitive to electron radiation and the measurement technique does apply electron radiation.
